# Genomic Landscape of Spitzoid Neoplasms Impacting Patient Management

**DOI:** 10.3389/fmed.2018.00344

**Published:** 2018-12-13

**Authors:** Lisa M. Hillen, Joost Van den Oord, Milan S. Geybels, Jürgen C. Becker, Axel zur Hausen, Véronique Winnepenninckx

**Affiliations:** ^1^Department of Pathology, GROW School for Oncology and Developmental Biology, Maastricht University Medical Center, Maastricht, Netherlands; ^2^Laboratory for Translational Cell and Tissue Research, Department of Pathology, KU Leuven, Leuven, Belgium; ^3^Department of Epidemiology, GROW School for Oncology and Developmental Biology, Maastricht University, Maastricht, Netherlands; ^4^Institute for Translational Skin Cancer Research, German Cancer Consortium (DKTK), Partner Site Essen, University Hospital Essen, Essen, Germany

**Keywords:** spitzoid neoplasms, spitz nevi, malignant spitz tumor, atypical spitz tumor, spitz melanoma, molecular, genetic, patient management

## Abstract

Spitzoid neoplasms are a distinct group of melanocytic proliferations characterized by epithelioid and/ or spindle shaped melanocytes. Intermediate forms that share features of both benign Spitz nevi (SN) and Spitz melanoma, i.e., malignant Spitz tumor (MST) represent a diagnostically and clinically challenging group of melanocytic lesions. A multitude of descriptive diagnostic terms exist for these ambiguous lesions with atypical Spitz tumor (AST) or Spitz tumor of uncertain malignant potential (STUMP) just naming two of them. This diagnostic gray zone creates confusion and high insecurity in clinicians and in patients. Biological behavior and clinical course of this intermediate group still remains largely unknown, often leading to difficulties with uncertainties in clinical management and prognosis. Consequently, a better stratification of Spitzoid neoplasms in benign and malignant forms is required thereby keeping the diagnostic group of AST/STUMP as small as possible. Ancillary diagnostic techniques such as immunohistochemistry, comparative genomic hybridization, fluorescence *in situ* hybridization, next generation sequencing, micro RNA and mRNA analysis as well as mass spectrometry imaging offer new opportunities for the distinct diagnosis, thereby allowing the best clinical management of Spitzoid neoplasms. This review gives an overview on these additional diagnostic techniques and the recent developments in the field of molecular genetic alterations in Spitzoid neoplasms. We also discuss how the recent findings might facilitate the diagnosis and stratification of atypical Spitzoid neoplasms and how these findings will impact the diagnostic work up as well as patient management. We suggest a stepwise implementation of ancillary diagnostic techniques thereby integrating immunohistochemistry and molecular pathology findings in the diagnosis of challenging ambiguous Spitzoid neoplasms. Finally, we will give an outlook on pending future research objectives in the field of Spitzoid melanocytic lesions.

## Introduction and Background

**Figure 1:**
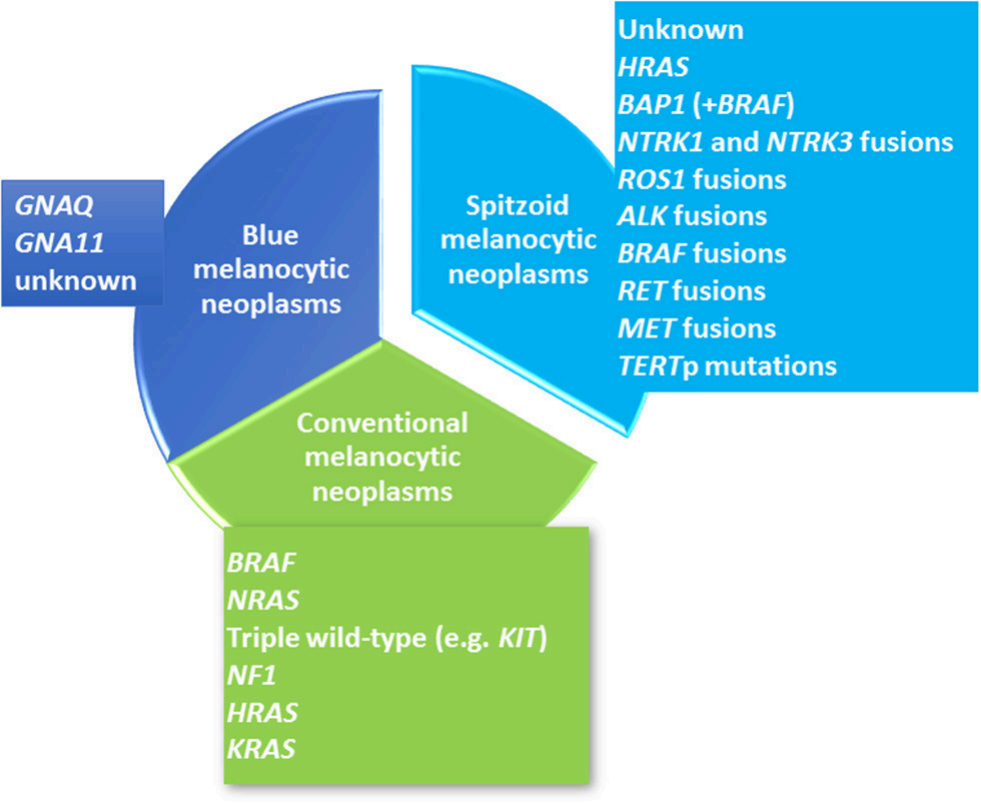
Overview of the Molecular Landscape of Melanocytic Neoplasms. Cutaneous melanocytic neoplasms can be clustered into three major lines including conventional melanocytic neoplasms (colored in green), Spitzoid melanocytic neoplasms (colored in turquoise) and blue melanocytic neoplasms (colored in blue). Each group shows distinct molecular genetic alterations. Conventional melanocytic neoplasms (green color) include common melanocytic nevocellular nevi, congenital nevocellular nevi and cutaneous malignant melanoma. Common nevocellular nevi harbor activating hotspot *BRAF* mutations at codon 600 [~80%, (1)]. The majority of congenital nevocellular nevi harbor activating *NRAS* hotspot mutations [~75%, (2)]. Cutaneous malignant melanoma has been clustered into four molecular subtypes (3) with the largest subgroup harboring a *BRAFV600E* mutation (~50%) and the second largest group with an activating *NRAS* mutation (~25%) as well as *HRAS* and *KRAS* mutations (~1%). The third subgroup shows *NF1* inactivation (~10%). The last molecular subgroup lacks *BRAF, N/H/KRAS* or *NF1* mutations and represents a heterogeneous so called “triple wild-type subtype”. The second major line of cutaneous melanocytic neoplasms comprises blue melanocytic neoplasms which are also called dermal dendritic melanocytic neoplasms (blue color) and have been shown to harbor activating *GNAQ* and *GNA11* mutations. *GNAQ* dominates in cutaneous proliferations while *GNAQ* and *GNA11* is found in almost the half of uveal melanomas (4). The third group refers to Spitzoid melanocytic neoplasms (turquoise color) and shows quite different molecular genetic alterations which are rarely observed in the two other groups. Receptor tyrosine kinase translocations involving *ALK, ROS1, NTRK1, NTRK3, BRAF, RET, MET* (5–7) as well as *HRAS* (5, 8, 9), *BAP1* (10), and *TERT*p mutations (11–13) have been described in Spitzoid neoplasms.

Melanocytic neoplasms comprise a heterogeneous group of tumors that are characterized by their distinct histopathological appearance, their clinical features and their genetic aberrations. Roughly, melanocytic neoplasms can be distinguished into three major groups of (i) conventional melanocytic neoplasms including benign acquired and congenital nevocellular nevi and cutaneous malignant melanoma, (ii) dendritic, i.e., blue melanocytic neoplasms and (iii) Spitzoid melanocytic neoplasms. Each group is characterized by a distinct histomorphological appearance what is also reflected by the spectrum of underlying genetic aberrations (Figure 1). In the group of conventional melanocytic neoplasms, which by far comprises the largest group, a clear-cut distinction between a benign nevocellular nevus and a malignant melanoma is usually possible on histological grounds.

According to the Cancer Genome Atlas (TCGA) cutaneous malignant melanoma comprises four molecular subtypes, i.e., melanomas with *BRAF V600E* mutation, melanomas with *NRAS* activating mutation, those with *NF1* mutation and the “triple wild-type” lacking mutations in the afore mentioned genes [Figure 1, (3)]. The second melanocytic group comprises blue melanocytic neoplasms which are characterized by a proliferation of dendritic, spindled and/ or ovoid dermal melanocytes and therefore are also named dermal dendritic melanocytic neoplasms (14). They show mutations in *GNAQ* and *GNA11* [Figure 1, (4)]. The third group of melanocytic neoplasms encompasses Spitzoid lesions revealing distinct histological features which set them apart from other melanocytic neoplasms. Of interest, this is also reflected in their underlying diverging molecular genetic profile [Figure 1 (15)].

**Figure 2:**
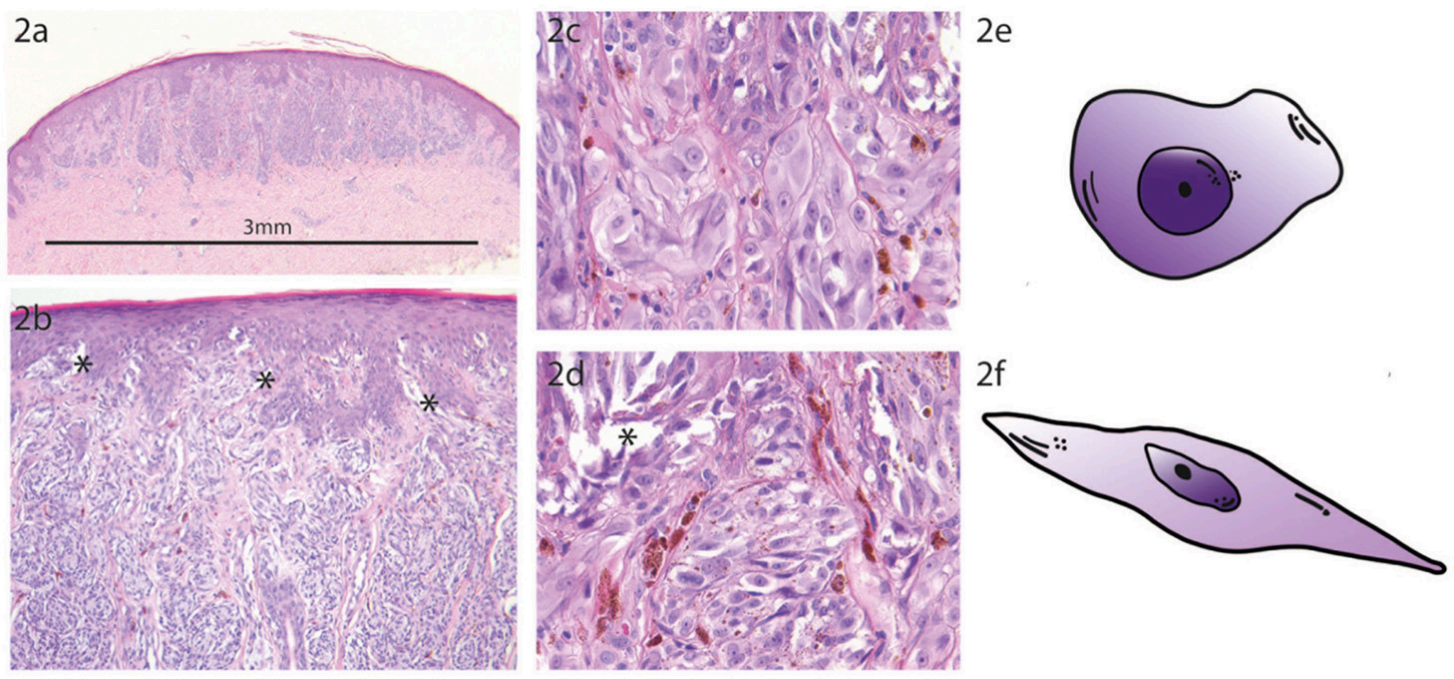
Prototypic Spitz Nevus. **(a)** The compound Spitz nevus (SN) has a small size of 3 mm with a symmetric dome shaped morphology and shows sharp lateral demarcation (H.E. staining, magnification factor 2.5x). **(b)** Higher magnification from A shows regular epidermal hyperplasia with vertically orientated fascicles of spindled and epithelioid melanocytes. Note the numerous epidermal junctional clefts (*) which are formed due to discohesion of the melanocytes (H.E. staining, magnification factor 10x). **(c)** Higher magnification from B with uniform characteristic epithelioid melanocytes at the epidermal junction (H.E. staining, magnification factor 40x). **(d)** Higher magnification from B showing regular spindle shaped melanocytes at the epidermal junction with formation of junctional clefts (*) due to discohesion of the Spitzoid melanocytes (H.E. staining, magnification factor 40x). **(e)** Schematic sketch of a prototypic epithelioid shaped melanocyte which is characteristic for a SN. **(f)** Schematic sketch of a prototypic spindle shaped melanocyte which is characteristic for a SN.

Spitzoid neoplasms are composed of large epithelioid and/ or spindle-shaped melanocytes with large nuclei that contain vesicular chromatin and often prominent nucleoli and are usually arranged in a distinctive architectural configuration (Figure 2). Spitzoid neoplasms represent uncommon melanocytic lesions and account for only about 1% of resected melanocytic neoplasms (16–18). The American pathologist Sophie Spitz was the first, who described them in 1948 as “juvenile melanomas” or as “melanomas of childhood” because they often appear in children or adolescents and show lymphotropic behavior with spread to locoregional lymph nodes (19). However, these lesions can also occur later in life and therefore were renamed as Spitz nevi (SN) to indicate their benign nature (20, 21). The term Spitz melanoma or malignant Spitz tumor (MST) was reserved to Spitzoid neoplasms with marked atypia and an aggressive clinical course eventually leading to metastasis and death. In daily surgical pathology practice Spitzoid melanocytic neoplasms often lead to diagnostic difficulties because they can reveal conflicting histomorphology intermediate of SN and MST. In these ambiguous cases morphology is often incapable to predict biological potential and thereby clinical outcome [(22), see case 12]. The diagnostic difficulties might stem from the idea that the morphological criteria which are applied for conventional melanocytic neoplasms also apply for Spitzoid melanocytic neoplasms. However, it should be considered that the group of blue melanocytic neoplasms, i.e., dermal dendritic melanocytic neoplasms is well recognized to have their own morphological criteria (14). Thus, it is quite likely that Spitzoid melanocytic neoplasms also have peculiar morphological criteria with their exact definition and nomenclature remaining an ongoing matter of debate and a pending area of future research (14, 22). The apparent diagnostic dilemma leads to high insecurity in daily practice with either under-diagnosis as benign SN or over-diagnosis as MST. Consequently, the diagnostic gray zone of ambiguous Spitzoid neoplasms has led to the introduction of a multitude of descriptive diagnostic terms such as atypical Spitz tumor (AST), Spitzoid tumor of unknown malignant potential (STUMP), melanocytic lesion of uncertain malignant potential (MELTUMP), superficial atypical melanocytic proliferation of uncertain significance (SAMPUS), dysplastic Spitzoid neoplasm, or borderline Spitzoid neoplasm. The multitude of these, often only vaguely defined descriptive histological terms hampers the ease of diagnostic and clinical work up, complicates interdisciplinary communication and increases uncertainty in treating dermatologists, surgical pathologists and last but not least in patients. Of note, the descriptive terms MELTUMP and SAMPUS are also used in the context of ambiguous non-Spitzoid melanocytic neoplasms that are characterized by conflicting criteria (14). In this review the term atypical Spitz tumor (AST) will be used for lesions exhibiting a Spitzoid morphology but also harboring features associated with malignancy according to the recently launched WHO 2018 classification of skin tumors (14). In this review we follow a three-tiered classification comprising the benign SN-, the intermediate AST- and the malignant MST category (14).

The unclear etiology and pathogenesis of Spitzoid neoplasms as well as the diagnostic difficulties has led to numerous additional studies aiming to better understand pathogenesis and to identify biomarkers for use in daily diagnostics.

In this review, we will briefly describe the histopathological features of Spitzoid neoplasms and give a detailed overview of the current knowledge of the molecular genetic landscape of Spitzoid neoplasms. We also discuss the usefulness and the limitations of ancillary methods in ambiguous Spitzoid neoplasms to achieve a more accurate and distinct diagnosis. A reasonable integration of histopathology and stepwise performance of ancillary immunohistochemical and/ or moleculargenetic techniques should aim to minimize the gray zone in between SN and MST thereby improving diagnostic accuracy and patient management. Finally, we will give an outlook on potential future research questions and unresolved research objectives in the field of Spitzoid melanocytic lesions.

## Histopathological Features of Spitzoid Neoplasms

The diagnosis of a Spitzoid neoplasm is primarily based on histopathological grounds. However, the diagnosis should always be made in the context of the clinical features, especially including the patient's age with lesions in adults harboring a higher suspicion for malignancy [Table 1, first row, (24)]. Spitzoid neoplasms may be junctional, compound or intradermal. Intradermal localization is mainly seen in adults (18, 25, 26). The most distinct feature of a Spitzoid neoplasm is the presence of enlarged epithelioid and/ or spindle-shaped melanocytes [Figures 2C–F, (16, 27)]. In addition to cell type other histopathological features are required to render the diagnosis of a Spitzoid neoplasm which are briefly summarized in Table 1.

**Table 1:** Clinical and histopathological features of spitzoid neoplasms.

	**Spitz nevus (SN)**	**Atypical Spitz tumor (AST)**	**Malignant Spitz tumor (MST), Spitz melanoma**
**CLINICAL FEATURES**			
Patient Age	Usually <20	Any, but usually >10	Any, but usually >10
Location	Any with limbs, face and/or neck most common	Any	Any
**ARCHITECTURE**			
Diameter	<5 mm^*^ (see also Figure 2a)	>5 mm	often >10 mm
Outline	Dome, symmetric, wedge-shaped^*^ (see also Figure 2a)	Asymmetric	Asymmetric
Circumscription	Sharp^*^	Often poor	Poor
Epidermal hyperplasia	Present (see also Figures 2a,b)	Often epidermal effacement	Absent or epidermal effacement
Maturation with dermal depth and “zonation”	Present^*^	Uncommon, absent	Usually absent
Subcutan involvement	Orderly at the deep margin^*^	Frequent subcutaneous extension with “pushing” margins	Irregular extension at the depth with infiltrative growth
**CELLULAR MORPHOLOGY**			
Cellular population	Uniform spindle and/or epithelioid^*^ (see also Figures 2c–f)	Spindle and/ or epithelioid cells with increasing cytologic atypia	Spindle and/or epithelioid cells with increasing cytologic atypia and pleomorphism
Cytoplasm	Opaque/ground glass	Granular	Granular/mixed
Nuclei and nucleoli	Open, delicate chromatin pattern, uniform nucleoli	Heterogeneous chromatin pattern, increasingly prominent nucleoli	Loss of dispersed chromatin pattern, hyperchromasia, large nucleoli
Nuclear/ cytoplasmic ratio	Low	Increasingly high	High
Pigment	Superficial distribution	Variable	Variable, deep and irregularly distributed
**PROLIFERATIVE ACTIVITY**			
Mitotic rate	Absent or rare, < 2/mm^2^ No atypical mitoses ^*^	2–6/mm^2^ Deep or marginal dermal mitoses may be present	2–6/mm^2^ Deep or marginal dermal mitoses frequently present
Proliferative index (MIB-1/ Ki-67 IHC expression)	< 2%	2–10%	>10%
**MISCELLANEOUS DIAGNOSTIC FEATURES**			
Ulceration	Absent	Often	Frequent
Kamino bodies	Present^*^	Absent or few	Typically absent
Host response	Inconspicuous	Fibroplasia; patchy mononuclear lymphocytic infiltrates in papillary dermis	Band-like, patchy
Other	Junctional clefts (see also Figures 2b,d), teleangiectasia, transepidermal elimination of cell nests		

Summary of histomorphological characteristics in the diagnosis of Spitzoid neoplasms with

* *highlighting most helpful diagnostic features in the differential of SN, Spitz nevus; AST, atypical Spitz tumor and Spitz melanoma, i.e., MST, malignant Spitz tumor. Adapted from the WHO 2018 classification (14) and Barnhill (23)*.

A SN is usually >5 mm in diameter, shows a dome or wedge shaped symmetrical outline with sharp lateral circumscription, maturation, and zonation to the depth of the lesion and epidermal hyperplasia [Figure 2A, (18, 26, 28)]. There should be no ulceration, absence or only random cytonuclear atypia, no involvement of subcutaneous tissue and no or only few mitoses (Table 1). Zonation of cells with large cells in the superficial layers and maturation of melanocytes from the surface to the depth with decrease in amount of cell nests, cell size, nuclei, and nucleoli is seen in benign SN in contrast to their atypical or malignant counterparts (16, 27). However, the frequency of maturation in Spitzoid neoplasms varies considerably among published studies (16, 18, 21). The distribution of pigment, if present, may be zonal as well and localized subjacent to the epidermis. Kamino bodies constitute an important diagnostic feature of Spitzoid neoplasms and favor a benign nature of the lesion (17, 29, 30). Ultra-structurally Kamino bodies are composed of amorphous filaments and they stain for basement membrane components, including collagen types IV, VII, and laminin on immunohistochemistry [IHC, (15, 31, 32)]. They do not show evidence of apoptosis (33) and should not be confused with Civatte or colloid bodies. Single-cell upward spread of melanocytes is less common in Spitzoid lesions compared to conventional melanocytic malignant neoplasms (18). If pagetoid spread of Spitzoid cells is present, it most often occurs in bundles or nests of cells. At the dermoepidermal junction, the fascicles of Spitzoid cells often show vertical orientation with loss of cohesion and form retraction spaces from the adjacent epidermis somehow resembling “bundles of bananas” or a “raining down” pattern; a feature often found in Spitzoid lesions and less common in melanoma (27). SN often show adnexotropism with involvement of hair follicles and eccrine ducts what should not raise undue concern about melanoma (34). Additionally the distribution of the inflammatory infiltrate in SN can be a helpful diagnostic feature, which is quite often perivascular and diffusely in between the collagenous material (27). In SMM the inflammatory infiltrate is usually found at the base of the lesion.

AST present with intermediate histological features of SN and MST. AST show increase in at least one worrisome histological feature i.e., ulceration, size >5 mm, infiltrative growth into subcutaneous tissue with “pushing” margins, increased cytonuclear atypia, increase in cell density with confluent growth, >2 dermal mitoses, absence of junctional clefts, few or no Kamino bodies and more extensive pagetoid extension (Table 1, second column). However, these histological features to distinguish AST from SN or SMM have often shown to be ineffective due to poor reproducibility (22). Therefore, assessment of an ambiguous Spitzoid neoplasm requires ancillary diagnostic techniques such as IHC as well as molecular pathology analysis, which will be outlined and discussed in the following.

Finally, MST is diagnosed on the basis of the following histological criteria: ulceration, asymmetrical architecture, infiltrative growth, severe and/ or confluent cytonuclear atypia, dermal mitoses, pushing borders, epidermal effacement and pagetoid extension (Table 1, last column).

## Underlying Molecular Alterations and Ancillary Studies in Spitzoid Neoplasms

The aim of many additional studies has been to identify criteria that enable assignment of Spitzoid lesions to a correct and distinct diagnosis of SN or MST and thus to keep the ambiguous diagnostic category of AST as small as possible. The major interest of these studies was to identify parameters that predict malignant behavior in Spitzoid neoplasms with increased risk of recurrence, risk for metastasis, and risk for death. In the following, the findings from IHC studies in Spitzoid neoplasms including cell cycle and apoptosis regulator related markers as well as application of melanocytic markers will be outlined. Recent findings from next generation sequencing (NGS) which have identified telomerase reverse transcriptase promotor (*TERT*p) mutations, *BRCA1* associated protein 1 (*BAP1*) mutations, *HRAS* mutations and receptor tyrosine kinase alterations (*ALK, ROS, NTRK1, NTRK3, RET, MET*) will be discussed. Furthermore, results from molecular pathology analysis using comparative genomic hybridization (CGH) and fluorescence *in situ* hybridization (FISH) will be reviewed and their diagnostic applicability will be discussed. We also discuss recent findings from mRNA and micro RNA (miRNA) expression studies. Finally, we will briefly focus on recent results from mass spectrometry imaging (MSI), which potentially represents a promising adjunct technique. The additional diagnostic techniques and molecular genetic targets which have been reported in the literature for Spitzoid neoplasms are summarized in Table 2. Their usefulness for the differential diagnosis of AST vs. MST or AST vs. SN is commented in the second last column of Table 2.

**Table 2:** Synopsis of ancillary investigations and molecular genetic targets reported in the literature in SN, AST and MST.

**Marker/ gene/ chromosome**	**Common investigation technique**	**Spitz nevus (SN)**	**Atypical Spitz tumor (AST)**	**Malignant Spitz tumor (MST)**	**Helpfulness in the differential AST vs. MST**	**References**
**CELL CYCLE AND APOPTOSIS REGULATORS**						
MIB-1/ Ki-67 (Chr. 18q11)	IHC	Junctional nuclear expression or in superficial dermis; no expression in the base of the lesion (< 2% of melanocytes)	Similar pattern as SN but higher expression index (2–10%)	High and diffuse expression throughout the lesion (>10%)	Helpful	(35–40)
PHH3 (Chr. 17q25)	IHC	Similar pattern as MIB-1/ Ki-67	Similar pattern as MIB-1/ Ki-67	Similar pattern as MIB-1/ Ki-67	Helpful	(41)
Cyclin D1 (Chr. 11q13)	IHC	Higher expression than MIB1/ Ki-67	Data unclear	Data unclear	Unclear	(42–44)
**MELANOCYTIC MARKERS**						
HMB45 (antibody against gp100, Chr. 12q13.2)	IHC	Cytoplasmatic staining limited to upper part of the lesion	Data unclear, NA	Expression persists deeply, Note: 25% of metastases are negative for HMB45	Of limited help	(45–47)
S100 (Chr. 1q21)	IHC	Weaker staining than melanoma	Data unclear, NA	Strong or weak staining possible	Not helpful	(48, 49)
S1006A (Chr. 1q21)	IHC	Diffuse expression	Diffuse expression	Patchy expression	Unclear, limited data	(49)
**OTHER MOLECULAR GENETIC TARGETS IN SPITZOID NEOPLASMS**						
P16 (Chr. 9p21) or Chromosome 9p loss	IHC and FISH	Strong nuclear and cytoplasmatic staining, but also heterogenous staining profiles	Data unclear, NA	Negative, complete loss is rare but probably a significant indicator of malignancy	Can be helpful	(50, 51)
P53 (Chr. 17p13)	IHC	Low nuclear staining index	Data unclear, NA	High nuclear staining index, late stage loss of p53	Unclear, limited data	(52)
P21 (Chr. 6p21)	IHC	High expression	High expression	Unclear, limited data	Unclear, limited data	(40)
*BAP1* (Chr. 3p21)	IHC, NGS	No loss	11% with *BAP1* inactivation	Unclear	Might be of some value in a subset of Spitzoid lesions together with *BRAFV600E* mutations	(10, 53–57)
*TERT*p (Chr. 5p15.33)	Sequencing	Absent	Can be present	Can be present	Controversial, limited data	(11, 12)
*HRAS* (Chr. 11p) or Chromosome 11p gains	FISH, NGS	Mutation or copy number increase in 15–20%	Mutation or copy number increase in 15–20%	Not detected	Can be helpful	(5, 8, 9, 58–61)
**TYROSINE KINASE FUSIONS**						
*ALK* (Chr. 2p23.2-p23.1) *NTRK1* (Chr. 1q23.1) *NTRK3* (Chr. 15q25.3) *MET* (Chr. 7q31.2) *RET* (Chr. 10q11) *ROS1* (Chr. 6q22.1) *BRAF* (Chr. 7.) *NRAS* (Chr. 1p13.2)	NGS, for some targets IHC and/ or FISH available	Fusions in up to 55% of SN	Fusions in up to 56% of AST	Fusions in up to 39% of MST	Not helpful; Kinase fusions are probably responsible for initiating tumorgenesis in Spitzoid neoplasms but do not distinguish benign from malignant lesions	(5–7, 56, 62–65)
**CHROMOSOMAL ABERRATIONS**						
Chromosomal gains	CGH, FISH (chromosomal loci in bold are available as FISH probe)	**11p gain** (15% of cases)or **7p, tetraploidy**	1p36 3p26 3q11 **6p25** 8q11 **8q24** 9p24 9q12 **11q13** 20p13	8p23 9q12 **11q13** 11q14	Helpful. It should be considered that CGH is largely limited to research centers whereas FISH is more affordable, but also less sensitive and specific.	(66–71)
Chromosomal losses	CGH, FISH (chromosomal loci in bold are available as FISH probe)	Usually none	1p13 1q21 3p11 4p16 5q12 **6q23** 7q21 8p21 9q24 9p12 **9p21** 14q11 18q11	**6q23** **9p21** 9p24 9q24		
**RNA ALTERATIONS**						
Micro RNA (miR)	Nanostring nCounter expression profililng, real time PCR	Upregulation of miR22 Downregulation of miR-125b and miR-211	Upregulation of miR-21 and miR-155	Upregulation of miR-21, miR-150 and miR-155	Limited Data, might be of potential help in the future	(72–74)

*CGH, Comparative Genomic Hybridization; FISH, Fluorescence in situ Hybridization; IHC, Immunohistochemistry; NA, not available; NGS, Next Generation Sequencing; PCR, Polymerase Chain Reaction*.

### Cell Cycle and Apoptosis Regulators

Assessment of IHC expression of the nuclear protein MIB-1/ Ki-67, which is upregulated by cells in the active phase of the cell cycle represents a widely used ancillary technique in the diagnosis of Spitzoid neoplasms (35–40, 42, 75). In SN IHC expression for MIB-1/Ki-67 is largely restricted to the upper parts of the lesion. These findings contrast with MST, in which MIB-1/Ki-67 expression is generally higher and also present in the depth of the lesion (Tables 1, 2). Phosphohistone-H3 (PHH3) IHC reflects mitotic activity and has also been studied in melanocytic lesions (41). The expression of PHH3 is more sparse compared to MIB-1/ Ki-67 because PHH3 IHC expression is restricted to mitotic figures from early prophase through metaphase, anaphase, and telophase whereas MIB-1/Ki-67 stains the whole replicative cycle including G1- and G2 phase. There are only few data for Cyclin-D1 IHC in Spitzoid lesions (42–44). Unlike in conventional malignant melanoma there seems to be a dissociation between Cyclin D1 overexpression and cell proliferation in Spitzoid neoplasms (43). Therefore, Cyclin D1 IHC is not recommended as adjunctive test in the diagnostic work up of Spitzoid neoplasms.

Mutations in p16 (syn. cyclin dependent kinase inhibitor 2A, *CDKN2A*) which is localized on Chr. 9p21 have been associated with an aggressive clinical behavior in AST (76). FISH analysis for *CDKN2A* represents an adjunctive tool (77) and loss of 9p21 is associated with malignant behavior in Spitzoid neoplasms. Although p16 IHC is of added value in the differential diagnosis of ambiguous Spitzoid neoplasms, its expression alone is not sufficient to discriminate between SN, AST, and MST (50, 51, 77). The use of p16 IHC to approach p16 mutational status in Spitzoid lesions remains to be interpreted with caution, because p16 is often heterogeneously expressed in these lesions and the customized antibodies for p16 IHC are not mutation specific.

### Melanocytic Markers

Spitzoid melanocytic neoplasms have been evaluated with a variety of melanocytic markers including HMB45, S100, S100A6, MITF, Mart-1, Melan A, and SOX10. They are widely used to establish melanocytic differentiation and are helpful to better assess the growth pattern in cases where melanocytes are poorly identifiable on H.E. histology. HMB45 (antibody against Gp100) is a melanogenesis related protein and useful in the assessment of a lesions maturation toward the depth. HMB45 IHC shows cytoplasmatic staining and diminishes in the depth of a SN, whereas in MST positivity has been described to be trans-lesional and more uniform without limitation to the upper parts of the lesion (45, 47). For AST the expression pattern is not very well described in the literature and seems to be of limited additional value in the differential diagnosis in between AST and MST. S100 IHC shows diffuse expression in Spitzoid melanocytic neoplasms independent from benign or malignant behavior and thus has not been proven as helpful in the differential diagnosis of ambiguous Spitzoid lesions (48, 49). The S100 subtype S100A6 has been proposed as potential IHC marker in the differential diagnosis of SN from MST with strong expression in SN and diminished expression in malignant lesions (49), but conformational studies are pending to validate its usefulness in the routine examination of Spitzoid neoplasms.

### Intracellular Signal Receptor Molecules and Receptor Tyrosine Kinase Fusions

The *HRAS* gene is located on the short arm (p) of chromosome 11 and copy number gains of 11p as well as activating *HRAS* mutations are found in approximately 20% of Spitzoid neoplasms (5, 8, 58, 78). This is in contrast to conventional melanoma where *HRAS* mutations are found in >1% of cases (3). Mutations in *HRAS* usually occur in exon 3, causing replacement of the glutamine at amino acid position 61 with a lysine (*HRASQ61K*) or an arginine (*HRASQ61R*). Mutations in exon 2 are infrequent. The amino acid exchange leads to constitutive activation of the MAP/ERK and the PI3K/AKT/mTOR pathway. It has been described that tumors with 11p gains often show an intradermal localization, a desmoplastic morphology with stromal sclerosis and infiltrative margins; proliferative activity is low and most often the prognosis is favorable (5, 59). Currently, it is not known why *HRAS* mutations occur almost exclusively in Spitz tumors. Wiesner et al. suggested that *HRAS* mutations lead to stronger activation of the PI3K/AKT/mTOR pathway than *BRAF* mutations do in common nevi, a feature that might explain why the melanocytes have a larger cell size in Spitz tumors with *HRAS* mutations (56). Although the current data are derived from a limited number of cases with only limited long term follow up the results suggest that *HRAS* mutations in Spitzoid lesions can be attributed to a benign biological behavior.

*BRAF* and *NRAS* mutations are commonly found in conventional melanoma [~70%, (79)] but are rare in Spitzoid lesions. Initial studies did not report *BRAF* and/ or *NRAS* mutations in SN (5, 80–82), while later studies detected *BRAF* mutations in up to 20% and *NRAS* mutations in up to 5% of Spitzoid neoplasms (5, 6, 60, 62, 83–85).

Fusions such as translocations of the receptor tyrosine kinases including *ALK, ROS1, NTRK1, NTRK3, RET*, and *MET* are found in up to 50% of Spitz tumors and are infrequent in conventional melanocytic lesions with *BRAF* mutations (6, 56, 64, 65).

*ALK* gene fusions are found in 10% of Spitzoid neoplasms (6, 64). The most prominent fusion partners are *TPM3* and *DCTN1* and a distinct morphology has been attributed to them with the majority being amelanotic, dome-shaped and with presence of plexiform intersecting melanocytic fascicles (63, 86). Spitzoid neoplasms with *ALK* alterations are often localized on the extremities (64). Recently, Farah and colleagues reported a case of a plexiform MST with *ALK* copy number gain instead of *ALK* rearrangement (87). In summary, *ALK* mutations appear to be associated with a characteristic plexiform growth pattern, but seem to be present in the whole spectrum of Spitzoid lesions. Therefore, alterations in *ALK* appear to be of limited help in distinguishing benign from malignant Spitzoid lesions.

*ROS1* gene fusions are observed in 17% of Spitzoid neoplasms, and often also occur on the extremities (6). Wiesner et al. found Spitzoid neoplasms with *ROS1* fusions to show a similar morphology as those with *ALK* fusions (63). As according to *ALK* gene fusions, *ROS* gene fusions are also found in benign and malignant Spitzoid neoplasms. Therefore, they seem to be of limited value to discriminate benign and malignant lesions.

Fusions of the neurotrophic receptor tyrosine kinase (*NTRK*) 1 gene have been described in up to 16% of Spitzoid neoplasms spanning a spectrum from benign to malignant (6). No distinct morphology has been linked to *NTRK1* rearranged Spitzoid lesions. These fusions have also been confirmed by IHC analysis (6). Another group found *NTRK3* fusions in Spitzoid neoplasms with a higher incidence in AST (6/8 cases) than in SN [2/8 cases, (7)].

Wiesner et al. described *RET* gene fusions in >5% of Spitz tumors. In analogy to other receptor tyrosine kinase fusions the MAPK/ERK and PI3/AKT/mTOR pathways are activated (6). Studies in mice have shown that *RET* overexpression results in melanocytic proliferation, nevus formation and progression to malignant melanoma (88). Since *RET*-positive Spitzoid neoplasms are rare, only sporadic data is available on the predictive value with regard to biological and clinical behavior.

*MET* gene fusions activate the MAPK/ERK and PI3K/AKT/MTOR pathways and have been described in six Spitzoid neoplasms in a study from (65).

In summary, the above described kinase gene fusions (including *ALK, ROS, NTRK1, NTRK3, RET*, and *MET*) occur in a mutually exclusive pattern and are not found in *BAP1* (see below) or *HRAS* mutated Spitzoid lesions (56). Furthermore, if present they are detected across the entire spectrum of Spitzoid lesions [i.e., in 55% of SN, 56% of AST and in 39% of MST, (6)], thereby suggesting that the fusions occur early in the pathogenesis of the tumors. Hence, diagnostic applicability in the differential diagnosis in between a benign or a malignant Spitzoid neoplasms seems to be of limited value (56, 57, 62, 84).

### *BRCA1* Associated Protein 1 (*BAP1*)

*BAP1* is a deubiquitinating enzyme and its inactivation is found in approximately 5% of Spitzoid neoplasms (10). The *BAP-1* mutated lesions have been called “Wiesner tumor” or “BAPoma” and often also show *BRAFV600E* mutations, which is not typical in Spitzoid lesions (10, 53, 54). IHC showing loss of nuclear *BAP1* expression seems to be a good surrogate marker for *BAP1* inactivation (10).

*BAP1* mutations can be associated with a hereditary tumor predisposition syndrome (OMIM #614327) and lesions often show a histologically distinct epithelioid phenotype (6, 10, 55). It has been described that tumors with sporadic *BAP1* loss have the same histological appearance as those seen in patients with germline *BAP1* mutations. Although infrequent, inactivation of *BAP1* together with *BRAFV600E* mutations might predict a benign clinical course in Spitzoid lesions (6, 53, 57, 64). However, it remains to be considered that the to date available data are based on studies with a limited number of cases with lack of long term follow up data and require conformational studies.

### Telomerase Reverse Transcriptase Promotor (*TERTp*)

Upregulation of *TERT*p in order to overcome shortening of telomers and consequently senesce has been reported in Spitzoid lesions (11, 12). Mutations in *TERT*p have been found in subgroups of AST and MST and were associated with an aggressive clinical behavior and distant metastasis (11). However, data are conflicting with other groups that report no association with poor prognosis in Spitzoid lesions (12). More studies are needed to better evaluate the usefulness of *TERT*p mutations in the differential diagnosis of AST from MST.

### Chromosomal Investigation Techniques

CGH is a molecular technique that analyses entire genome copy number alterations by comparing tumor DNA to normal reference DNA. SN usually lack copy number alterations except for gains in 11p (attributed to *HRAS* activation) in desmoplastic SN (9, 78, 89) and very rarely isolated loss of chromosome 3 in epithelioid melanocytic tumors with *BAP1* loss (10). Mutational activation in *HRAS* does not occur in other nevi and is extremely rare in conventional melanomas (58, 90). As mentioned above *HRAS* activation in Spitzoid neoplasms might point to a benign behavior of the lesions (58).

AST reveal more genomic aberrations than SN, but fewer than MST. The identification of copy number alterations with CGH has evolved into FISH assays, which are less labor intensive and require only basic laboratory equipment. The most extensively studied FISH assay comprises 4 loci including chromosome 6 with the *RREB1* region (6p26), chromosome 6 with the *MYB* region (6q23), centromere 6 (Cep6) and/ or chromosome 11 with the *CCND1* region (11q13), and/or chromosome 9 with the *CDKN2A* region [9p21; (66, 91, 92)]. The so called “melanoma FISH” which targets 6p26, 6q23, Cep6, and 11q13 shows high sensitivity and specificity in unequivocal conventional melanocytic neoplasms (66, 93), but was less reliable in cases of Spitzoid melanocytic neoplasms (67). Adaptation of chromosomal targets with probes against 9p21, 11q13, and 8q24 (targeting the *c-MYC* region) has led to improvement in the diagnosis of equivocal Spitzoid neoplasms (66, 68, 70). Table 2 summarizes the currently identified chromosomal targets in Spitzoid neoplasms. It has been shown that homozygous deletions of 9p21 and/ or gains in 6p26 and or 11q13 are associated with a worse clinical behavior in Spitzoid neoplasms whereas deletions in 6q23 favor a benign behavior (67, 70).

Although at first sight FISH analysis seems to be more cost saving than CGH it is important to note that FISH analysis of melanocytic lesions has a comparatively high false negative rate. With FISH analysis only 4–6 genomic loci are investigated, and mutations, small insertions or deletions as well as genomic rearrangements cannot be detected. Additionally, FISH can also show false positive results due to tetraploidy in Spitz nevi occuring in 5–10% of SN (94). Tetraploidy is seen in melanocytes when they are stimulated to divide and replicate their DNA but subsequently do not divide as a result of a halting of the process by regulators of the cell cycle. Hence, the cells are trapped and typically undergo senescence. In summary, tetraploidy can be seen in conventional melanocytic neoplasms and/ or Spitzoid neoplasms and is therefore non-diagnostic. Thus, the diagnostic utility of FISH analysis in the evaluation of equivocal Spitzoid neoplasms remains a subject of intense discussion (69, 95).

### miRNA Expression Profiling

Until now only few studies have investigated the miRNA profile of Spitzoid melanocytic tumors (72–74). MiRNA represent 19–22 nucleotide, non-coding RNA molecules that epigenetically regulate gene expression at the post-transcriptional level by either degradation or translational blockade of target mRNA (96). Deregulation of miRNA with oncogenic and tumor-suppressive functions has attracted much attention and it has been shown that the expression profile of miRNA varies across neoplastic lesions at different stages of malignancy (97, 98).

Grignol et al. investigated the expression of miR-21, miR-155, and miR-211 in conventional malignant melanoma and borderline melanocytic lesions (including 22 AST samples) with real-time PCR and compared them to benign common nevi (72). The conventional malignant melanoma group and the borderline melanocytic lesion group (including the AST group) showed upregulation of miR-21 and miR-155 when compared to benign nevi (72). SN or MST samples were not investigated in this study. Latchana et al. have identified distinct miRNA expression profiles in the whole spectrum of Spitzoid melanocytic neoplasms including SN, AST, and MST with the NanoString nCounter Gene Expression platform (73) and investigated a selection of 12 reversely transcribed miRNAs by real time PCR (73). In their study benign SN showed decreased expression of miR-125b and miR-211, and upregulation of miR-22, compared to benign nevi (74). MST showed overexpression of miR-21, miR-150, and miR-155 when compared to benign nevi and also when compared to the AST group. Summarizing, these studies indicate that upregulation of miR-21 and miR155 might be attributed to a malignant behavior in Spitzoid melanocytic neoplasms (72, 74). Summarizing, the analysis of miRNA expression represents a promising additional approach to the categorization of indeterminate Spitzoid melanocytic lesions and may be used as a diagnostic adjunct or eventually therapeutic target in the future.

### mRNA Expression Profiling

Characterization of the transcriptome of Spitzoid melanocytic neoplasms by mRNA sequencing has gained increasing attention in the last years (13, 99, 100). Wu et al. analyzed the landscape of fusion transcripts in metastatic MST and biologically indeterminate AST with positive locoregional lymph nodes by RNA sequencing (13). They successfully investigated six out of seven samples and identified novel fusion transcripts involving *TPM3-NTRK1* (2 samples), rearrangements in *TPM3, ALK* and *IL6R* (1 sample), *BAIAP2L1-BRAF* (1 sample), *EML4-BRAF* (1 sample), and two samples containing a second fusion gene, *ARID1B-SNX9* or *PTPRZ1-NFAM1*. Two tumor samples with metastatic disease harbored a *TERT*p mutation which was also confirmed with mRNA *in situ* hybridization (ISH).

Jansen et al. investigated RNA expression differences in long intergeneic non-coding RNA 518 (LINC) and preferentially expressed antigen in melanoma (PRAME) from SN and compared them to conventional malignant melanoma (100). From the 23 investigated samples nine samples comprised pediatric SN. While PRAME showed significantly higher expression in malignant melanoma cases in relation to SN cases, LINC expression was not different in both groups. AST or MST were not investigated in this study.

Our group recently investigated the mRNA expression profile of SN in comparison to common nevocellular nevi (99). A significant difference in the mRNA signature in between these two groups was shown even when SN and nevocellular nevi samples were obtained from the same individuals. We could identify a molecular genetic signature of 15 top ranked genes with upregulation of *CAPN2, CDC25B, ITGA3, LIG4, HSPA6, DUSP10*, and *JAK3* and downregulation of *IGFR1, IDH1, HES1, BAIAP3, SUV39H2, SHC2, FGF2*, and *MSH6* transcripts in SN when compared to NCN. We are currently expanding the mRNA expression analysis to AST and MST samples to investigate whether there is a differential mRNA expression profile in the whole spectrum of Spitzoid melanocytic lesions (SN vs. AST vs. MST). We are also evaluating whether the identified transcripts can be identified on translational level with immunohistochemistry.

Although no definitive conclusions can be drawn from the to date performed mRNA expression studies because of the limited number of studies and the relatively small numbers of investigated samples, the results from mRNA analysis are likely to reflect the actual functional state of the neoplastic cells and future studies should expand upon transcriptome sequencing in Spitzoid melanocytic neoplasms.

### Mass Spectrometry Imaging (MSI)

MSI is a powerful new methodology to analyze proteomic signatures in fresh frozen and also in formalin-fixed paraffin embedded (FFPE) tissue samples with spatial fidelity. Few studies have implemented MSI in order to assess its value in the differential diagnosis of melanocytic lesions. Hardesty and colleagues found peptides that discriminate prognostic subgroups in melanoma patients (101). Lazova and colleagues could discriminate SN with 97% sensitivity and 90% specificity from MST based on a mass spectral proteomic signature of five heterogeneously expressed peptides. The identified signature consisted of five distinct MSI peaks with the specific mass-to-charge (m/z) values 976.49, 1060.18, 1336.72, 1410.74, and 1428.77. For two of the five distinct values the corresponding peptides could successfully be identified, which are Actin (m/z = 976.49) and Vimentin (m/z = 1428.61). In a subsequent study the identified proteomic MSI signature could correctly classify 102 AST samples with an adverse clinical outcome to the MST signature and those with benign outcome to the SN signature (102). Interestingly, in a following study there was no detectable differential expression of Vimentin or Actin with IHC or immunofluorescence analysis (103) in between SN and MST samples. Although conformational studies are pending, these findings strongly suggest that there may be some characteristics on molecular level that are detected by MSI to better distinguish the gray zone AST category beyond the information provided by histopathology or immunohistochemistry.

## Diagnosis, Classification and Management of Spitzoid Neoplasms

**Figure 3:**
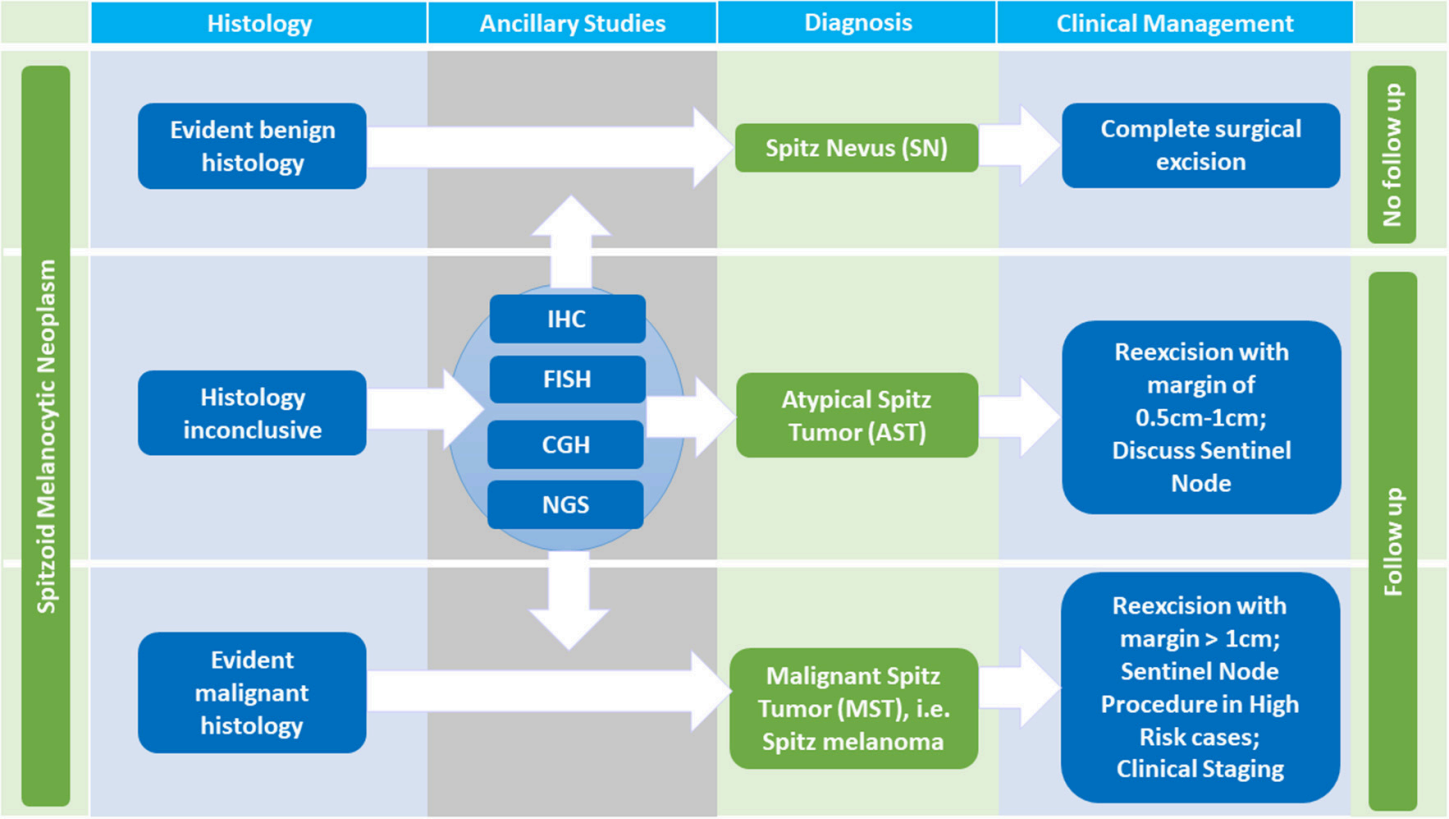
Diagnosis and Management of Spitzoid Melanocytic Neoplasms. Histopathologic evaluation is the initial step in diagnosing a Spitzoid neoplasm. In case of evident benign histologic characteristics, the diagnosis of a Spitz nevus (SN) can be made. If the lesion has been completely excised a clinical follow up is not necessary. In cases of evident malignant histopathology, the diagnosis of Spitz melanoma, i.e., malignant Spitz tumor (MST) is made and clinical management according to conventional malignant melanoma protocols should be followed. The diagnosis of MST necessitates a re-excision with a margin of at least 1 cm, in high risk cases a sentinel node procedure, clinical staging and follow up. If the histopathology is ambiguous stepwise ancillary studies such as immunohistochemistry (IHC), comparative genomic hybridization (CGH), fluorescence *in situ* hybridization (FISH) and next generation sequencing (NGS) should be performed. If histopathology as well as ancillary studies still remain inconclusive the diagnosis per exclusionem of atypical Spitz tumor (AST) is made. A reexcision with a margin of 0.5–1 cm should follow. The value of a sentinel node procedure, the follow up time, and the extent of clinical staging in this setting remains a topic of ongoing debate.

After all, the histopathological evaluation remains the initial step and gold standard in diagnosing a Spitzoid neoplasm (Figure 3, first column). Clinical features such as patient's age should always be considered with adults harboring higher suspicion for malignancy (24).

In cases of evident benign histomorphological characteristics with absence of any suspicious criteria (i.e., diameter < 5 mm, symmetrical architecture, sharp circumscription, maturation toward the dermal depth, no subcutan involvement, no striking cytonuclear atypia, no increase in proliferative activity with < 2 mitosis/mm^2^ and absence of ulceration, Table 1, first column) the diagnosis of a SN should be rendered. In patients diagnosed with SN a clinical follow up is not required if the lesion has been completely excised [Figure 3, first line, (104)]. If the excision shows positive margins on histopathological examination, we advise a re-excision or at least clinical follow up to monitor potential locoregional recurrence.

In cases of evident malignant histopathology, the diagnosis of MST is made and clinical management according to conventional malignant melanoma protocols should be followed with a re-excision comprising a margin of at least 1 cm, in high risk cases a sentinel node procedure, clinical staging, and follow up (Figure 3, third line).

**Figure 4:**
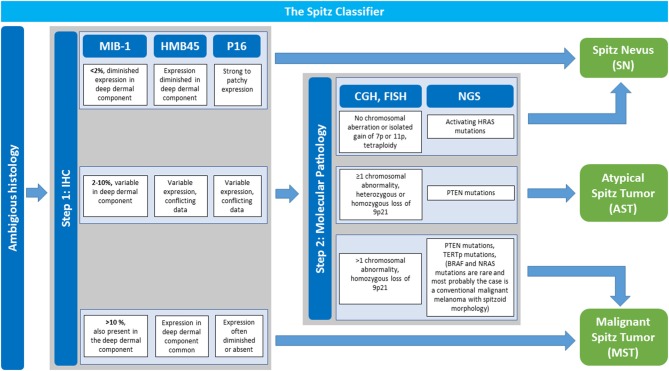
The Spitz classifier: Stepwise Implementation of Immunohistochemistry and Molecular Pathology in Ambiguous Spitzoid Neoplasms. In cases with ambiguous histopathological features that give suspicion for malignancy stepwise ancillary studies are performed to render the diagnosis of a Spitz nevus (SN), atypical Spitz tumor (AST) or malignant Spitz tumor (MST) with more security. Step 1 is immunohistochemistry (IHC) with a panel of MIB-1-, HMB45-, and p16 antibody. In dependence of the obtained result a diagnosis of SN or MST can be made. A MIB-1 IHC index < 2% with diminished expression in the deep dermal component as well as diminished HMB45 IHC expression in the deep dermal component and patchy to strong p16 IHC expression throughout the lesion favors the diagnosis of SN. Cases with MIB-1 IHC expression >10%, persisting HMB45 IHC expression in the deep dermal component as well as loss of p16 IHC expression favor the diagnosis of MST although exceptions of this rule are possible. If results of IHC investigation are intermediate with a MIB-1 IHC index of 2–10%, and/or variable HMB45- and p16 IHC expression one should proceed to step 2 with implementation of molecular pathology. Comparative genomic hybridization (CGH) analysis or if not available fluorescence *in situ* hybridization (FISH) are performed. Next generation sequencing (NGS) is of help as well. No detectable chromosomal aberration or isolated gain of Chr. 7p or 11p as well as activating *HRAS* mutation favor the diagnosis of a SN. The presence of at least one chromosomal abnormality (excluding Chr. 7p or 11p gain), especially homozygous loss of Chr. 9p11 as well as *PTEN* and *TERT*p mutations argue for MST. If the afore mentioned findings are only partly present with ≥1 chromosomal abnormality (including heterozygous and very rarely homozygous loss of Chr. 9p21), or *PTEN* mutations AST as a diagnosis per exclusionem is to be considered. Adapted from WHO 2018 classification (14).

If the histopathology is ambiguous one should not hesitate to ask for a second internal and/ or external consultation. In addition, ambiguous cases justify stepwise ancillary studies such as IHC, CGH, FISH, and NGS which are illustrated in Figure 4, of which IHC stands out as first step due to its technical ease and availability in pathology laboratories. An IHC panel including MIB-1/ Ki67, HMB45, and p16 can be useful [(14, 105, 106), Figure 4, see also Table 2 for details]. In special cases with a plexiform morphology *ALK* IHC as surrogate marker to address *ALK* gene rearrangement can be of help as well (86) and in cases with a distinct epitheloid phenotype *BAP1* IHC as surrogate for *BAP1* gene inactivation can be applied (10). A MIB-1 IHC index < 2% with diminished expression in the deep dermal component as well as diminished HMB45 IHC expression in the deep dermal component and patchy to strong p16 IHC expression throughout the lesion favors the diagnosis of SN. Cases with MIB-1 IHC expression >10%, persisting HMB45 IHC expression in the deep dermal component as well as loss of p16 IHC expression argue for the diagnosis of MST although exceptions of this staining pattern are possible.

If results from step one with IHC investigation are intermediate showing a MIB-1 IHC index of 2–10%, and/ or variable HMB45- and p16 IHC expression step two should be followed with implementation of molecular pathology (Figure 4). Since molecular pathology techniques such as CGH, FISH, and NGS are not always available in all pathology laboratories it is recommendable to refer the patients including histopathology tissue to a reference center in ambiguous cases. No detectable chromosomal aberration or isolated gain of Chr. 7p or 11p as well as activating *HRAS* mutation favor the diagnosis of a SN. The presence of at least one chromosomal abnormality (excluding 7p or 11p gain), especially homozygous loss of Chr. 9p11, loss of Chr. 6q23 as well as *PTEN* and *TERT*p mutations argue for MST.

It remains to be noted that interpretation of NGS results is an issue of current intense investigation and there exists no standardized NGS panel recommendation for Spitzoid neoplasms yet.

Investigation of the *BRAF* gen, either with the mutation specific *BRAFV600E* antibody or with sequencing analysis are of help in the differential diagnosis to which group the melanocytic lesion should be ascribed. Presence of a *BRAFV600E* mutation most likely rules out a Spitzoid melanocytic neoplasms and assigns the atypical lesion to the group of conventional melanocytic neoplasms. Thus, most probably the diagnosis would be a “conventional” melanoma harboring a Spitzoid epithelioid and- or spindle shaped morphology.

If the afore mentioned findings from molecular pathology investigation are only partly present with ≥1 chromosomal abnormality (including heterozygous and very rarely homozygous loss of 9p21), or *PTEN* mutations are present, AST as a diagnosis per exclusionem is to be considered. A patient with the diagnosis of the vaguely defined category AST should be managed with a re-excision with a margin of 0.5–1 cm and follow up is necessary (Figure 3, second line). The value of a sentinel node procedure, the extend of clinical staging and the exact follow up time in this setting remain unclear and a topic of ongoing debate (104, 107, 108). Unlike conventional melanoma, dissemination to regional lymph nodes in AST, in general, seems not to be predictive for distant metastasis or patient survival (104, 107–109).

**Figure 5:**
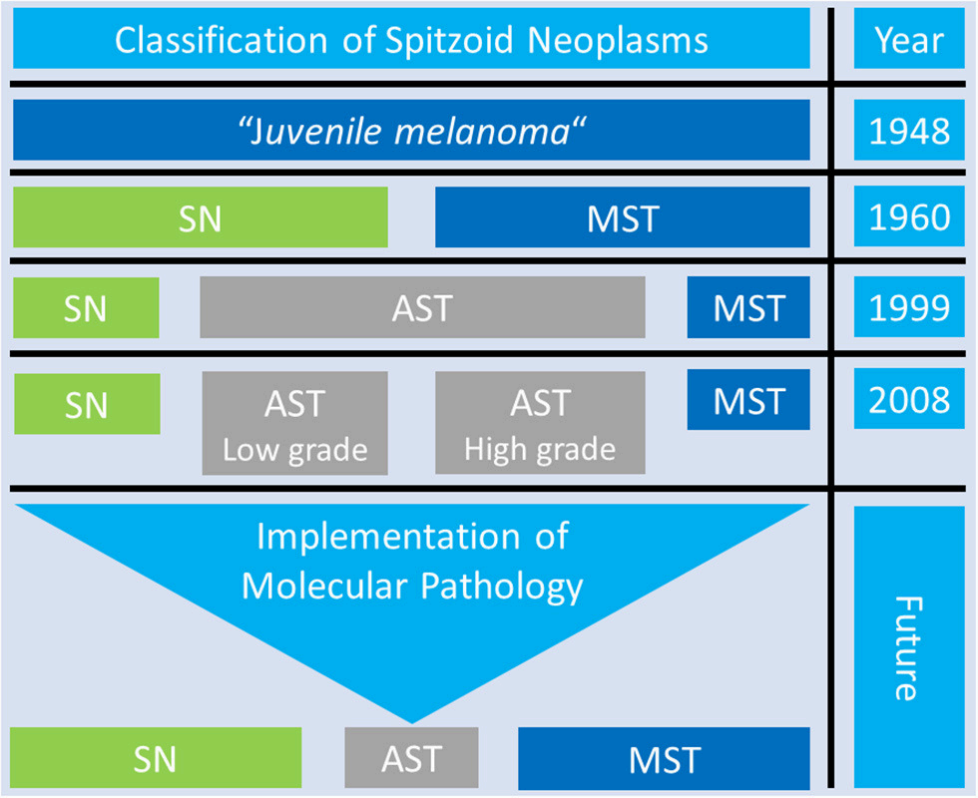
Overview of the Classification Systems of Spitzoid Neoplasms. Over time there have been many different diagnostic approaches to classify Spitzoid neoplasms. The proposed three- and four- tiered classification system best reflect the existence of the group op ambiguous Spitzoid neoplasms with atypical Spitz tumor (AST, gray color) with intermediate features in between complete benign Spitz nevi (SN, green color) and clear cut malignant Spitz melanoma, i.e., malignant Spitz tumor (MST, blue color). The application of ancillary diagnostic techniques and implementation of findings from molecular pathology might help to reduce the uncertain diagnostic category of AST to a minimum and improve and clarify patient management in the future.

Over time there have been numerous approaches to classify Spitzoid neoplasms which are summarized in Figure 5. The initial description “juvenile melanoma” by Sophie Spitz (19) was revised by Ackermann and colleagues who proposed a two-tiered classification system (20, 21, 106). This classification system differentiated between benign SN and MST as the malignant counterpart (Figure 5, third line). The ambiguous intermediate category of AST was not considered. From on the 1990s the two-tiered classification system was opposed by a three-tiered categorization including SN, AST and MST [Figure 5, fourth line, (22, 23)]. The international Melanoma Pathology Study group even suggested a four-tiered classification system which subclassifies AST in low grade and high grade lesions [Figure 5, fifth line, (84, 110)]. The proposed three- and four- tiered classification systems best reflect the existence of an ambiguous group of Spitzoid neoplasms with intermediate features in between complete benign SN and clear-cut malignant MST. Recently Tetzlaff and colleagues have emphasized the need toward a molecular based classification system for the morphologically ambiguous and biologically unpredictable group of AST [Figure 5, sixth line, (57)]. The application of ancillary diagnostic techniques and the implementation of the recent molecular genetic findings in the categorization of Spitzoid lesions is also suggested by the recently published WHO 2018 Classification of Skin Tumors (14) and might provide a framework to keep the uncertain diagnostic and therapeutic category of AST at a minimum thereby benefiting patient management.

## Future Research Objectives

The findings from the to date identified molecular alterations have not led to emergence of one stand-alone or a clearly defined distinct set of biomarkers that can reliably distinguish whether an ambiguous Spitzoid lesion will behave benign or malignant in the future. The urgent need remains to extend research projects and further immerge into the search of predictive biomarkers.

First, the analysis of the transcriptome and further proteomic studies, which are likely to represent the actual functional state of a cell, represent a promising strategy for identification of potential robust biomarkers. In first pilot studies the miRNA profile (72–74) and the mRNA profile (13, 99, 100) of Spitzoid melanocytic lesions have been investigated. The results are promising and might motivate surgical pathologists, clinicians and basic scientists to extend their investigations to this research field with larger study numbers and correlation of results with available long term follow up data.

Secondly, Spitzoid melanocytic neoplasms are well-known to harbor prominent collagenous or fibrotic stroma with fine vascularization and presence of infiltrating lymphocytes (16, 18, 111–113). Despite these obvious histological characteristics only little is known about the underlying immunological cellular subtypes, especially the nature of the infiltrating T-lymphocytes and the dermal stromal microenvironment in Spitzoid melanocytic lesions (18, 114). Even less is known about the microenvironment of the locoregional lymph nodes, which often harbor metastatic Spitzoid cells, but have been shown to be not predictive for aggressive biological behavior such as in conventional melanoma (104). We recently identified upregulated gene pathways in SN with increased expression of mRNA transcripts that were related to immunomodulatory, inflammatory, and extracellular matrix interactions as well as angiogenesis associated processes (99). Others have identified a proteomic discriminatory MSI signature, which is based on the stromal environment around SN and MST (115, 116). One may speculate that the immunological and stromal microenvironment might be a potential discriminator in the differential diagnosis of AST vs. MST. We hope that these observations and the preliminary findings will stimulate future research efforts to better characterize the dermal and lymphonodular microenvironment of Spitzoid melanocytic neoplasms.

Finally, the lack of objective and reproducible histologic criteria to determine the malignant potential of Spitzoid tumors remains a major diagnostic challenge. Very often the morphology does not reliably reflect the biological potential of a Spitzoid lesion. Apparently, the established histopathologic criteria which are used to differentiate nevocellular nevi from conventional malignant melanoma do not account for the group of Spitzoid melanocytic neoplasms (22, 117, 118). Thus, a critical revision of the to date applied histopathological criteria and the current classification of Spitzoid melanocytic neoplasms with uniformization of terminology is required. The recently launched WHO 2018 classification gives a first outline for a molecular based classification of Spitzoid melanocytic neoplasms (14) upon which diagnostic algorithmic approaches should be validated in the future.

## Conclusion

In summary, the diagnosis of a Spitzoid lesion requires accurate correlation of the clinical, histological and molecular features. Yet, there are no isolated clinical, histological or molecular criteria nor a clearly defined diagnostic pattern of criteria that can distinguish AST from SN or MST with adequate certainty (56, 57, 119–121). A proper differentiation between AST, SN, and MST remains one of the most challenging issues in the field of dermatopathology with each diagnosis having quite different consequences for patient treatment, clinical management and follow up. Rigorous characterization of sufficient numbers of Spitzoid neoplasms and long-term follow up is therefore necessary for the formulation of optimal guidelines for the care of patients with these lesions.

In conclusion, the results from the current ancillary techniques and molecular genetic findings should be interpreted together with the clinical and histopathological data to formulate a firm integrative diagnosis and to keep the uncertain diagnostic category of AST as small as possible.

## Author Contributions

VW, JV, and LH designed and developed the outline and concept of the manuscript. VW, JV, LH, and AzH supervised the writing of the manuscript and edited the figures and tables. LH, JV, MG, JB, AzH, and VW wrote, discussed and commented the manuscript. All authors approved the final manuscript.

### Conflict of Interest Statement

The authors declare that the research was conducted in the absence of any commercial or financial relationships that could be construed as a potential conflict of interest.
